# Bedside voluntary and evoked forces evaluation in intensive care unit patients: a narrative review

**DOI:** 10.1186/s13054-021-03567-9

**Published:** 2021-04-22

**Authors:** Djahid Kennouche, Eric Luneau, Thomas Lapole, Jérome Morel, Guillaume Y. Millet, Julien Gondin

**Affiliations:** 1 Laboratoire Interuniversitaire de Biologie de la Motricité (LIBM), Univ Lyon, UJM-Saint-Etienne, EA 7424, 42023 Saint-Etienne, France; 2 Département d’anesthésie et de réanimation, Centre Hospitalier Universitaire, Saint- Etienne, France; 3 Institut Universitaire de France (IUF), Paris, France; 4 Institut NeuroMyoGène (INMG); CNRS 5310 – INSERM U1217 - UCBL1; Faculté de Médecine et de Pharmacie, 8 Avenue Rockefeller, 69008 Lyon, France

**Keywords:** Intensive care unit-acquired weakness, Skeletal muscle function, Electrical stimulation, Magnetic stimulation, Ergometers

## Abstract

Around one third of intensive care unit (ICU) patients will develop severe neuromuscular alterations, known as intensive care unit-acquired weakness (ICUAW), during their stay. The diagnosis of ICUAW is difficult and often delayed as a result of sedation or delirium. Indeed, the clinical evaluation of both Medical Research Council score and maximal voluntary force (e.g., using handgrip and/or handheld dynamometers), two independent predictors of mortality, can be performed only in awake and cooperative patients. Transcutaneous electrical/magnetic stimulation applied over motor nerves combined with the development of dedicated ergometer have recently been introduced in ICU patients in order to propose an early and non-invasive measurement of evoked force. The aim of this narrative review is to summarize the different tools allowing bedside force evaluation in ICU patients and the related experimental protocols. We suggest that non-invasive electrical and/or magnetic evoked force measurements could be a relevant strategy to characterize muscle weakness in the early phase of ICU and diagnose ICUAW.

**Supplementary Information:**

The online version contains supplementary material available at 10.1186/s13054-021-03567-9.

## Introduction

Around one third of ICU patients will develop severe neuromuscular alterations, known as intensive care unit-acquired weakness (ICUAW), during their stay [1]. ICUAW, defined as “*a syndrome of generalized limb weakness that develops while the patient is critically ill and for which there is no alternative explanation other than the critical illness itself*” [2], is the most related disease acquired in ICU. Both persistent reduction of force production and atrophy are involved in the poor health-related quality of life [3]. There is so far no effective treatment to counteract the long-term deleterious effects of ICUAW [4]. This is mainly due to the difficulties of making an early diagnosis which further limit our knowledge of the underlying pathophysiological mechanisms of ICUAW [5]. ICUAW can be caused by (i) a critical illness polyneuropathy (CIP) which has been described as a distal axonal sensory-motor polyneuropathy affecting limb and respiratory muscles [6]; (ii) a critical illness myopathy (CIM) which is considered as a primary myopathy that is not related to muscle denervation [7]; (iii) a combination of both also referred to as critical illness polyneuromyopathy (CIPNM) [8].

ICUAW diagnosis currently relies on manual muscle testing using the Medical Research Council (MRC) score [9]. This method is easy to perform as it does not require any special equipment. Handgrip (HG) [10] and handheld (HHD) [11] dynamometers have been used to provide quantitative values of the patient’s maximal strength. However, both MRC score and voluntary force measurements can be obtained only in awake and fully cooperative patients so that ICUAW diagnosis is inevitably delayed.

To overcome these limitations, non-volitional techniques combining electrical [12] or magnetic [13] stimulation applied to a motor nerve or over a muscle belly [14] with force measurements on a dedicated bedside ergometer have emerged in the field of ICU. This allows to record evoked-force in fully sedated patients, so that measurement can be performed early after ICU admission (*i.e.* within 24 h of ICU admission [15]).

The aim of this narrative review is to summarize the different tools and related experimental protocols allowing bedside force evaluation of limb muscles in ICU patients. We will exclude the tools used to assess respiratory muscle function since it has been described in details elsewhere [5, 16]. After a brief presentation of the gold-standard method for ICUAW diagnosis (*i.e.* MRC scale), we will present for each existing dynamometers and ergometers: (i) intra- and inter-investigators reliability, (ii) their interests and limitations for quantitative voluntary and evoked-force measurements at the bedside, (iii) an overview of the cross-sectional studies in which force production was compared between ICU patients and healthy controls as well as longitudinal approaches in which the time course of changes in force was assessed throughout an ICU stay. The search strategy is described in Additional File 1.

## MRC scale

### Definition

MRC is currently the gold-standard diagnostic method of ICUAW. This scale includes the measurements of 6 muscle groups (shoulder abductors, elbow flexors, wrist extensors, hip flexors, knee extensors and ankle dorsiflexors) on each side, ranking them between 0 (no visible contraction) and 5 (normal force on complete range of motion). The MRC scale is an ordinal scale which gives a sum-score (*i.e.* sum of each individual muscle score) ranging from 0 (paralysis) to 60 (normal force).

### Reliability of MRC sum-score

Intra-investigator and inter-investigator reliability was considered as a test–retest performed by the same investigator on different occasions and measurements performed by different investigators on the same day, respectively. MRC reliability has mainly been assessed by intraclass correlation coefficients (ICCs), ranging between 0 (no agreement) and 1 (excellent agreement). So far, intra-investigator reliability of MRC has never been reported in ICU patients, even though good to excellent ICC values were observed in other pathologies [17, 18]. Inter-investigator reliability of the MRC sum-score was found to be good to excellent in ICU patients [19–23] (Table 1). However, lower ICC values (*i.e.* ranging from 0.29 to 0.75) were reported when considering individual muscle groups [20], indicating that MRC is less reliable to assess force from a single muscle group. High inter-investigator reliability was also reported for the four-point scale (ICCs = 0.90–0.94) on a small cohort of ICU patients [24].

**Table 1:** Inter-investigator reliability measurements of MRC sum-score

References	Number of patients	Number of investigators	ICC (95% CI—range)
Hermans et al. [19]	75	2	0.95 (0.92–0.97)
Hough et al. [20]	30	2	0.83 (0.67–0.93)
Kleyweg et al. [21]	60	2	0.97 (0.96–0.98)
Fan et al. [22]	10	19	0.99 (0.98–1.00)
Connolly et al. [23]	20	2	0.94 (0.85–0.98)

Intraclass Correlation Coefficients (ICC) and their 95% Confidence Interval (CI) are reported

### Diagnostic approach

**Fig. 1:**
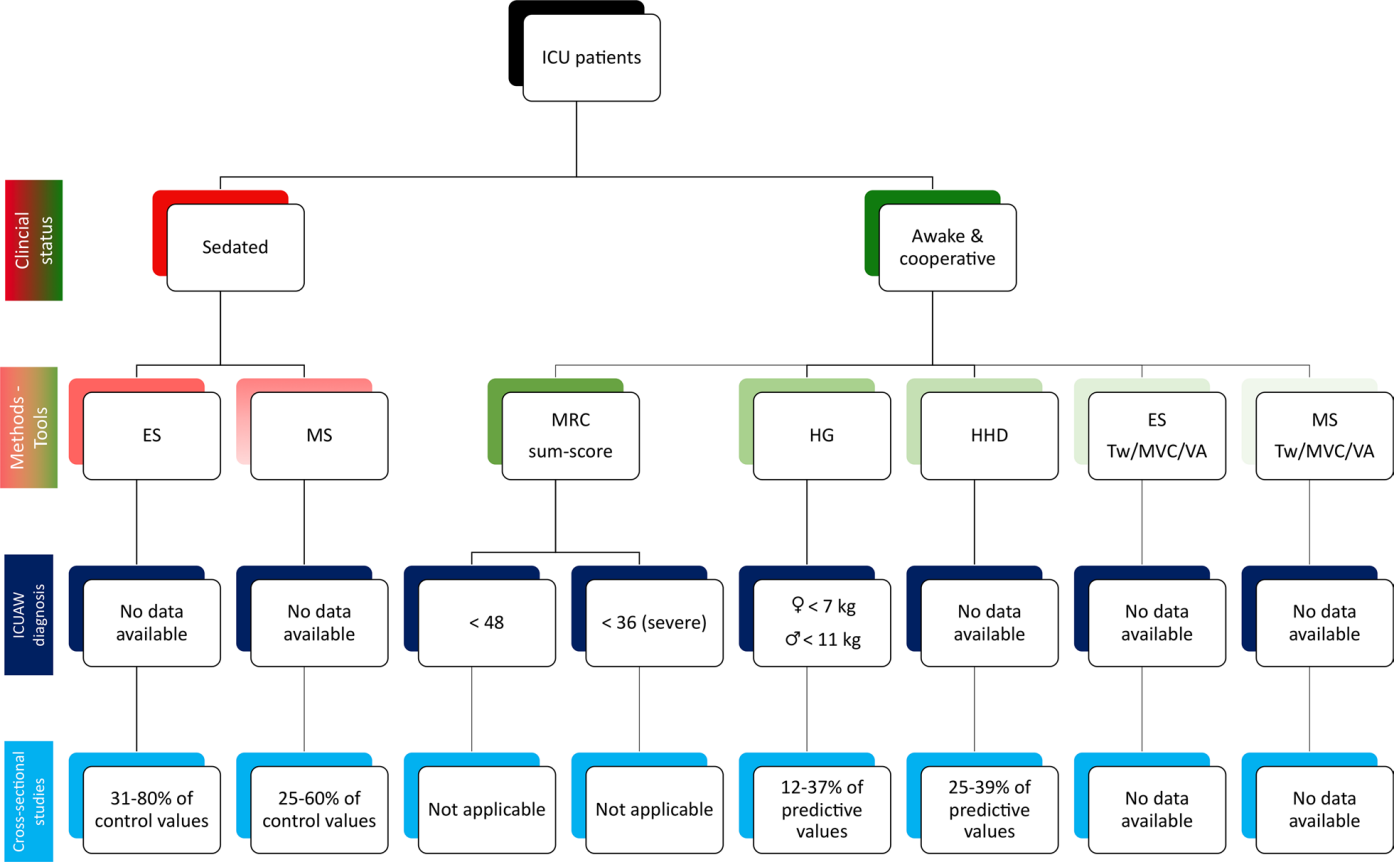
Overview of the different methods and tools used to diagnose intensive care unit-acquired weakness (ICUAW) and to quantify voluntary (with HG strength < 7 kg for females and < 11 kg for males) and evoked force in sedated and awake/cooperative ICU patients. ES: electrical stimulation; HG: Handgrip dynamometer; HHD: Handheld dynamometer; MRC: Medical Research Council; MS: magnetic stimulation; MVC: maximal voluntary contraction force; Tw: twitch; VA: voluntary activation (index of neural drive). All the data are derived from references reported in Tables 3 and 4 and are expressed as a percentage of values recorded in healthy subjects (*i.e.* control/predictive)

An MRC sum-score below 48 has been arbitrarily used for ICUAW diagnosis [25] and a score below 36 indicates severe muscle weakness [19] (Fig. 1). A four-point ordinal scale has been recently introduced but remains to be validated on a large cohort of patients [26]. An MRC sum-score < 40 has been proposed as a modality to specifically diagnose patients with CIPNM [27]. However, this study suffers from several limitations such as highly selective inclusion criteria (*i.e.* only septic patients), a small sample size (*i.e.* only 50 patients), a lack of information regarding the electrophysiological investigations and the day of assessment. Interestingly, it has been recently demonstrated that even a slight reduction in MRC sum-score (*i.e.* ≤ 55) at ICU discharge may identify patients with poor long-term outcomes (*i.e.* mortality, strength, functional capacity and physical function) [28]. Further studies are needed to determine whether and to what extent this specific cut-off MRC sum-score at ICU discharge is influenced by the etiology of ICUAW (*i.e.* CIP, CIM or CIPNM).

### Limitations

The first important limitation of MRC is that the grade ≤ 3 only uses gravity as reference whereas grade > 3 refers to muscle contraction against non-standardized and subjective resistance so that it is difficult to differentiate grade 4 (subnormal strength) and grade 5 (normal strength). MRC is further limited by a ceiling effect that precludes accurate measurements of strength. Although MRC sum-score is a good predictor of both hospital [29] and long-term [28] mortality, patients have to be awake and fully cooperative so that the diagnostic is inevitably delayed and can be further complicated by pain, edema or limitation on range of motion [30].

## Voluntary force measurements

### Existing ergometers and associated protocols

**Fig. 2:**
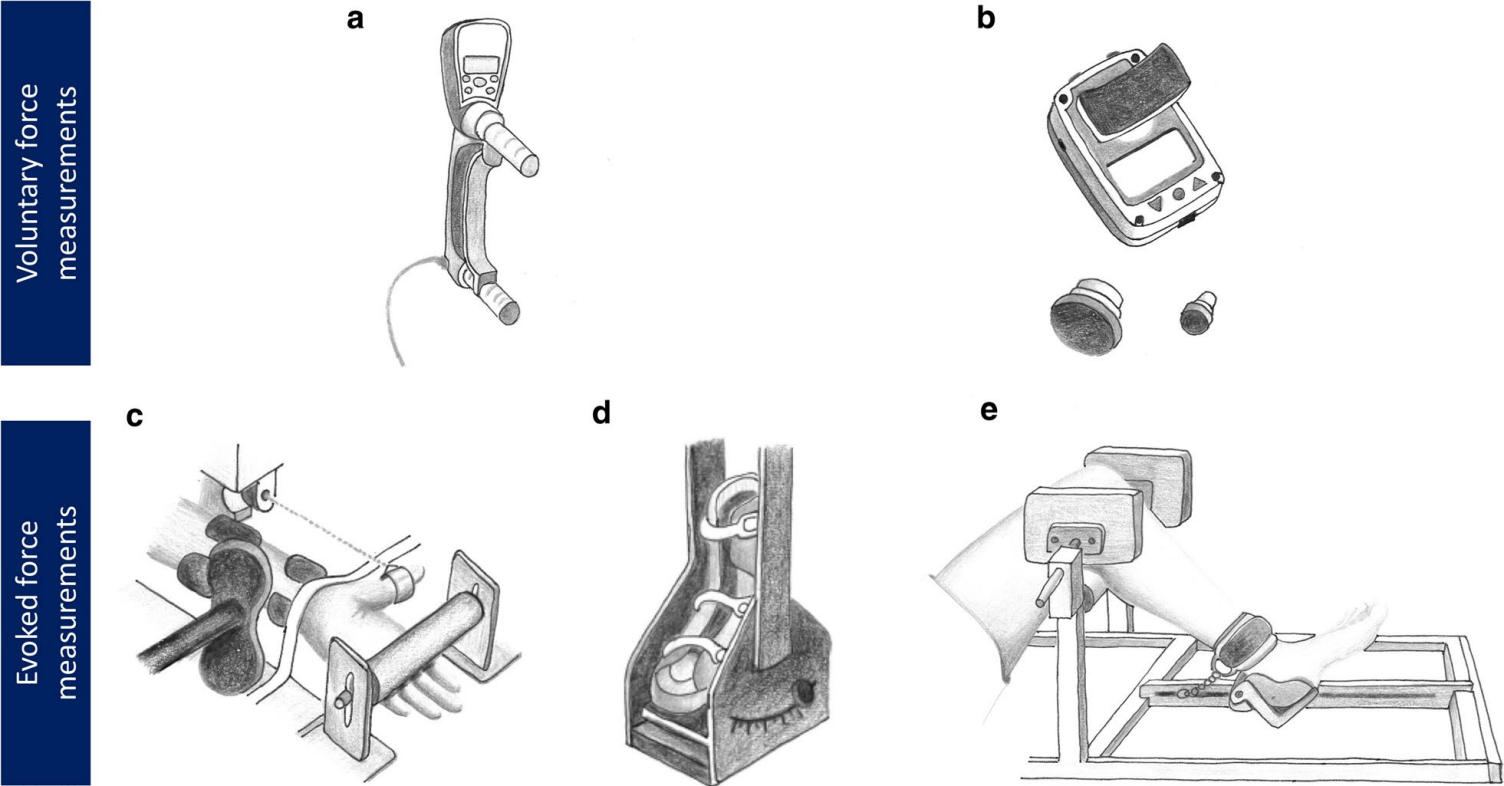
Typical handgrip (**a**) and handheld (**b**) dynamometers/ergometers used to record evoked-force on adductor pollicis (**c**, adapted from Harris et al. [13]), ankle dorsiflexors (**d**, adapted from Ginz et al. [56, 60]) and quadriceps (**e**, adapted from Laghi et al. [58])

HG dynamometer (Fig. 2a) has been used to provide quantitative strength values in ICU patients who are sufficiently cooperative and have an MRC score ≥ 3 in at least four of six muscle groups being tested [11]. Different experimental protocols have been used to quantify voluntary strength by HG, *i.e.* measurements are not fully standardized among studies in ICU patients [31]. A majority of studies followed the recommendations of the American Society of Hand Therapists by performing testing in a seated position [10, 19, 27, 32–37] while other investigations used a supine position to take into account the patients’ inability to maintain a stable vertical position [11, 24, 38–41]. Both shoulder and forearm were in neutral/rotation position when testing in seated position while the elbow joint angle varied from 90° of flexion to full extension when testing in supine position.

HHD (Fig. 2b) have been used to assess voluntary strength in several muscle groups from awakening to ICU discharge. Voluntary force measurements can be performed in both lower and upper limb muscle groups (*i.e.* wrist extensors, elbow flexors and extensors, shoulder extensors, abductors and rotators, hip flexors and extensors, knee extensors, ankle dorsiflexors) in less than 15 min [42]. As for HG, HHD does not require any complex training for the physician/investigator. The protocol is carried out in a supine position to avoid measurement errors due to gravity and to consider the inability of ICU patients to remain seated at the edge of the bed [39, 43]. Knee extension force measurements have been performed with either the test leg positioned over a bolster [39, 40] or at 30° [38] to 90° [11] of knee flexion. Considering these differences in testing position, methodological guidelines have been provided to standardize HG and HHD force measurements in ICU patients [11, 19, 39].

### Reliability

Intra-investigator reliability of HG measurements has been reported to be excellent in ICU patients (ICC = 0.86–0.92), although lower than that observed in healthy subjects (ICC = 0.97–0.99) [39]. Inter-investigator reliability was also good to excellent for HG in ICU patients (Table 2), as illustrated by the ICCs ranging from 0.88 to 0.97 [19, 24, 39]. Again, higher ICCs were found in healthy subjects (0.96 and 0.97) as compared to ICU patients (0.89 and 0.92) [39].

**Table 2:** Inter-investigator reliability of force measurements performed with handgrip and handheld dynamometers in ICU patients

References	Patients	Handgrip		Handheld					
		Right	Left	Shoulder abduction	Elbow Flexion	Wrist extension	Hip flexion	Knee extension	Ankle dorsiflexion
Vanpee et al. [11]	39			0.91 (0.85–0.95)	0.96 (0.93–0.98)	0.94 (0.91–0.97)	0.80 (0.67–0.89)	0.94 (0.90–0.97)	0.76 (0.33–0.90)
Hermans et al. [19]	46	0.93 (0.86–0.97)	0.97 (0.94–0.98)						
Parry et al. [24]	29	0.97 (0.90–0.99)^W^	0.94 (0.82–0.98)^W^						
		0.88 (0.70–0.96)^M^	0.97 (0.91–0.99)^M^						
Baldwin et al. [39]	15	0.92 (0.68–0.98)	0.89 (0.54–0.97)		0.71 (− 0.21 to 0.93)^R^			0.84 (0.52–0.95)^R^	
					0.62 (− 0.30 to 0.90)^L^			0.79 (0.34–0.93) ^L^	

Intraclass Correlation Coefficients (and their 95% Confidence Interval) are reported

*W* women, *M* men, *R* right, *L* left

Excellent intra-investigator reliability of HHD measurements was obtained in all muscles but the left elbow flexors (ICC = 0.42). This later finding could be explained by the presence of radial arterial lines during the initial measurements [39]. Inter-investigator reliability of HHD was good to excellent [11, 39] (except for the elbow flexors, Table 2). However, Vanpee et al. [11] reported higher ICCs for elbow flexion and knee extension as compared with those measured by Baldwin et al. [39]. This could be related to several methodological differences between the two studies, such as the definition of awakening, the testing position, the use of visual feedback during force measurements, the age of the patients, the length of stay at the first force assessment and the experimental design.

### Voluntary force in ICU patients: cross-sectional, longitudinal and diagnostic approaches

#### Cross-sectional approach

Absolute HG forces largely varied between studies and ranged from 3 to 20 kg during the first week of admission [32, 36, 44] and from 6 to 16 kg [19, 24, 32, 39–41] over the second and third week (Table 3). Absolute HHD forces ranged from 8 to 12 kg for knee extension and elbow extension after a median ICU stay of 13–16 days [39, 40]. Several factors can explain the large differences among studies. Both age and the proportion of men could influence absolute force values [24, 45]. Older patients (> 80 years old) had lower HG absolute force values than their younger counterparts after ICU discharge [45]. It has also been reported that women had a median HG score of 0 kg while men obtained a median score of 20 kg when measurements were performed around 9 days after awakening [24]. In addition, the longer the mechanical ventilation, the lower the absolute grip force values (Table 3) [32]. Severity of illness (*i.e.* APACHE III) was also higher and HG force was lower in patients with ICUAW as compared with patients without ICUAW [10].

**Table 3:** Main outcomes from studies measuring voluntary force with handheld (HHD) and handgrip (HG) dynamometers in ICU patients

References	Number of patients	% Men	Age (years)	Ventilation duration (days)	ICU LOS (days)	Disease severity	Scoring system	Method	Testing session (days)	Main results
Ali et al. [10]	35 with ICUAW 101without ICUAW	40 50.5	59.5 57.1	12 6	21 12	66	APACHE III	HG (seated)	N/A	Handgrip cutoffs values for ICUAW diagnostic: 7 kg for women, 11 kg for men
Vanpee et al. [11]	39 + 12 (retest)	62	64	N/A	N/A	N/A	N/A	HHD	N/A	Absolute force for shoulder abduction: 74 N; elbow flexion: 75–79 N; wrist extension: 61–62 N; hip flexion: 112–119 N; knee extension: 85–94 N; ankle dorsiflexion: 57–80 N No gender difference for force loss
Hermans et al. [19]	46	59	N/A	N/A	15	N/A	N/A	HG (seated)	15	HG absolute force for right hand: 11 kg for women *vs* 19 kg for men
Parry et al. [24]	60	58	69	6.6	12	22	APACHE II	HG (supine)	9	HG absolute median force values were 20 kg and 0 kg in men and women, respectively 27% of patients had a grip force of 0 kg (majority of women with n = 14/16)
Schmidt et al. [27]	28 with ICUAW 22 without ICUAW	39 50	58 49	N/A	40 14	77 66	APACHE IV	HG (seated)	N/A	Definition of new HG cutoff values (4 kg and 7 kg in women and men, respectively) to diagnose ICUAW as compared with EMG measurements HG absolute force values were lower in patients with ICUAW as compared with patients without ICUAW (2.5 *vs* 13.6 kg, respectively)
Cottereau et al. [32]	3 groups: 41 33 10	37 60 10	58 68 69	4 11 20	8 17 24	47 52 60	SAPS II	HG (seated)	First SBT: 4 8 12	Absolute HG force values were of 20; 12 and 6 kg at day 4; 8, and 12 of SBT, respectively Relative HG force values were of 30; 29 and 25% at day 4; 8, and 12 of SBT, respectively (normative database of Bohannon et al. [48])
Borges et al. [33]	72	36	53	7.5	10	20	APACHE II	HHD (quadriceps) + HG (seated)	Hospital discharge	Quadriceps and HG relative values were: 51% and 55% (Normative database of Hogrel et al. [65])
Bragança et al. [34]	45	60	55	5	10	69	SAPS III	HG (seated)	N/A	ICUAW patients had lower HG absolute force values as compared with patients without ICUAW (4 *vs* 22 kg, respectively) HG cutoff values [10] had high agreement with MRC criteria for ICUAW diagnosis
Sidiras et al. [35]	36 with ICUAW 92 without ICUAW	42 74	58 51	18 08	26 12	18 15	APACHE II	HG (seated)	ICU and hospital discharge	ICUAW patients are weaker than patients without ICUAW at both ICU and hospital discharge (3 *vs* 14 kg and 7 *vs* 16 kg, respectively) Women had lower HG relative force values at ICU and hospital discharge as compared with men
Borges & Soriano [36]	37	54	53	5	10	56	SAPS III	HG (seated)	3 and hospital discharge	HG absolute force values were of 12 and 19 kg at day 3 and hospital discharge, respectively HG relative values were of 37% *vs* 68% at day 3 and hospital discharge, respectively (normative database of Günther et al. [46]
Burtin et al. [38]	90	72	57	N/A	N/A	25	APACHE II	HHD + HG (supine)	ICU and hospital discharge	No significant difference for quadriceps force (normalized to body weight) measured with HHD between ICU and hospital discharge: 1.86 N.kg^−1^ and 2.03 N.kg^−1^
Baldwin et al. [39]	17	59	78	10	18	20	APACHE II	HHD + HG (supine)	13	HG, elbow flexion and knee extension absolute force values for right side were: 11; 9 and 11 kg, respectively
Baldwin & Bersten [40]	16	56	62	13	20	94	APACHE III	HHD + HG (supine)	16	HG, elbow flexion and knee extension absolute force values for right side were 11; 9 and 8 kg, respectively
Chlan et al. [41]	120	49	52	N/A	N/A	61	APACHE III	HG (supine)	9 (still ventilated)	Mean HG force was 3.2 kg (ranging from 0 to 54 kg) with 6 patients having a force of 0 kg No force improvement was observed over time under MV
Dietrich et al. [45]	253	52 50	< 80 > 80	8 6.5	10 9	14	APACHE II	HG (seated)	1–5 after ICU discharge	HG absolute force (dominant, non-dominant): 20 and 18 kg for patients < 80 years *vs* 15 and 13 kg for patients > 80 years
Samosawala et al. [49]	64	64	49	N/A	9.6	N/A	N/A	HHD	3; 5 and 7	Absolute force decreased by 11.8% between day 3 and 7
Morris et al. [50]	300	45	56	N/A	7.5	76	APACHE III	HHD + HG (seated)	ICU and hospital discharge	HHD and HG absolute values at ICU and hospital discharge were: 9.9 kg and 10.4 kg *vs* 20.9 and 24.3 kg, respectively
Segaran et al. [51]	44	78	58	4–16	7–16	20	APACHE II	HG (seated)	N/A	HG measurements not feasible due to a lack of alertness (assessed by 4 questions), weakness and poly-trauma

*EMG* electromyography, *LOS* length of stay, *HHD* handheld dynamometer, *HG* handgrip, *ICU* intense care unit, *ICUAW* intense care unit-acquired weakness, *MV* mechanical ventilation, *NO* no ICUAW, *SBT* spontaneous breathing trial; (II), (III), (IV): Score acute physiology and chronic health evaluation II, III, IV; SAPS: Simplified acute physiology scores II, III. Median values are underlined

To take into account these differences, HG and HHD force has been normalized to age- and sex-matched values recorded in healthy controls to obtain relative/predictive values, *i.e.* values that patients should get if they had no ICUAW. Relative HG force values ranged from 12 to 37% of predicted values during the first week of ICU stay [32, 36, 44] (Fig. 1). This heterogeneity can also be due to differences in duration of MV and ICU stay and disease severity. Surprisingly, the lowest relative force values (12% of predicted values) were observed in a specific cohort of patients admitted to surgical ICU and having a very short MV (median of 3 days) and ICU stay (median of 5 days) duration [44]. This unexpected result could be related, at least in part, to the fact that 55% of the patients had HG force of 0 kg [44].

HG, elbow flexion and knee extension relative force values ranged from 25 to 39% of predicted values (Fig. 1) and from 51 to 54% in the late phase of ICU admission (12–16 days) [32, 40] and at hospital discharge [33, 35, 36], respectively. Interestingly, patients with septic shock displayed lower predictive values than patients with only severe sepsis [33], illustrating the impact of disease severity on force production. Despite the use of relative/predictive values, differences in the clinical status of patients and in the normative database [46–48] clearly limit the comparison of voluntary force measurements among studies in ICU patients.

#### Longitudinal approach

To circumvent the aforementioned issues, longitudinal voluntary force measurements have been performed with both HG and HHD during ICU stay [41, 49]. HG force remained unchanged from day 1 to day 5 of awakening (*i.e.* the initial strength measurements were performed at a median ICU stay of 3 days) [36] or declined throughout the ICU stay (*i.e.* -0.34 lb-force per additional day of MV ventilation, especially in older women) [41]. Force recorded by HHD was reduced by 10–13% from day 3 to day 7 of ICU stay [49]. Altogether, these findings indicate that force does not recover within the first days of awakening.

Changes in voluntary force production between ICU and hospital discharge are still unclear. Indeed, knee extension force remained unchanged [38] while higher HG force values were reported at hospital discharge as compared with ICU discharge, although no statistical analysis was performed between the two measurements [35, 50]. ICUAW patients were always weaker than patients without ICUAW at both ICU and hospital discharge [35]. Further longitudinal studies are needed to determine the time course of voluntary force production from awakening to hospital discharge.

#### Diagnostic approach

The ability of HG to diagnose ICUAW has been investigated and cut-off values of less than 11 kg in men and less than 7 kg in women (Fig. 1) allowed to discriminate ICUAW with a sensitivity of 0.81 and a specificity of 0.83 [10]. In addition, MRC scores were positively correlated with HG force values. The use of HG as a surrogate to MRC to diagnose ICUAW was further confirmed by Parry et al. [24] with a sensitivity of 0.88 and a specificity of 0.80. However, the specificity was lower in women (0.45–0.55) than in men (0.88–0.92), despite a perfect sensitivity for women (*i.e.* 1.0). The lower specificity in women was explained by a very large number of women (14 out of 16) with a grip strength of 0 kg. Finally, the cut-off values of Ali et al. [10] were further confirmed in another population of ICU patients [34]. Lower cut-off values (*i.e.* 7 kg for men and 4 kg for women) were reported in septic patients diagnosed with CIPNM [27]. However, these results should be interpreted with caution considering the above-mentioned methodological limitations of this study [27]. Surprisingly, no study has investigated if HHD could be used to diagnose ICUAW despite its good to excellent reliability (Table 2 and Fig. 1).

### Limitations

Normative database of voluntary force vary among studies which limits the interpretation of cross-sectional studies. In addition, patients must be sufficiently awake/cooperative to be able to develop a voluntary force and should have an MRC score higher than 3 in elbow flexion and wrist extension [19]. However, criteria used to define awakening state have not been standardized between studies [11, 39]. In addition, HG force measurements had a significant floor effect with ~ 25–55% of patients having a score of 0 kg [24, 44, 51]. This could be related to the diagnosis of ICUAW [24], lack of alertness or motivation as well as fatigue and pain associated with the disease. Finally, the first voluntary force measurements are usually performed after a median ICU stay ranging from 3 [36, 44, 49] to 16 days [40]. In this context, non-volitional force measurements could be an attractive alternative to HG/HHD in order to provide an earlier characterization of neuromuscular function in sedated patients (Fig. 1).

## Evoked force

### Existing ergometers and associated protocols

Non-volitional force measurements can be obtained in sedated patient using an ergometer consisting of a force transducer and an adjustable platform combined with supramaximal stimuli applied either over a motor nerve or a muscle belly [52]. The most used technique is electrical stimulation (ES) [53] that usually allows to spatially recruit all motor units. Magnetic stimulation (MS), which consists in applying a magnetic field over a motor nerve through an ergonomic coil to depolarize motor axons, has been introduced as an alternative to ES [54] to limit discomfort. The main advantages of evoked force either with ES or MS rely on the possibility to assess muscle force in sedated patients in the very early phase of ICU admission (*i.e.* within 24 h of ICU admission, [15]). Evoked force can be obtained in response to single twitch (Tw), paired pulse and/or trains of stimulations [52]. So far, different ergometers have been developed allowing to record evoked force in response to ES and/or MS in ICU patients on three muscle groups (Fig. 2c–e): adductor pollicis [12, 13, 55], ankle dorsiflexors [15, 56] and quadriceps [14, 57, 58].

#### Adductor pollicis (AP)

The first ergometer to assess AP force in ICU patients was inspired by a device (Fig. 2c) previously developed by Merton [59]. Forearm and hand are immobilized in supinated position in an arm board. An adjustable metal loop connected to a strain gauge is positioned around the proximal phalanx of the thumb to record evoked force in response to stimulation applied over the ulnar nerve. The thumb is abducted and the metacarpophalangeal and interphalangeal joints are fully extended.

#### Ankle dorsiflexors

The ergometer used to assess the ankle dorsiflexors force in response to peroneal nerve stimulation (Fig. 2d) combines a boot fixed on an adjustable footplate with a strain gauge to record the evoked force [15, 56, 60]. The system can be adjusted to fit the patient’s leg that is firmly maintained using non-elastic straps. The patient is lying in bed, with the leg fully extended, the other one being maintained to avoid unwanted motion during the stimulation.

#### Quadriceps

Three different ergometers have been developed so far (one of them is shown on Fig. 2e), to record quadriceps force production in response to stimuli applied either over the femoral nerve [58, 61] or the muscle belly [14]. The ergometers have been designed to optimize knee and hip angle joint angles when patient is in supine position. The tested leg is positioned on a support and a strain gauge is attached next to the malleolus perpendicularly to the leg axis.

### Reliability

Reliability of evoked-force measurements has been scarcely investigated in ICU patients. No studies have measured intra- or inter-investigator ICC values and only a few investigations have reported coefficient of variations (CV) between two measurements. Intra-investigator reliability of magnetically-evoked Tw forces was found to be good in AP muscle (CV of 7.8% [3–9%]) [13]. Data extraction from Laghi’s et al. study [58] indicated an excellent intra-investigator reliability of quadriceps evoked force recorded in response Tw and paired pulse MS stimulations in ICU patients (CV of 1.9% [0.6–7.7%] and 1.5% [0.2–3.4%], respectively). In the same way, a good reliability of quadriceps Tw measurements in response to MS was also suggested on the basis of Bland–Altman comparison of values recorded in sedated and awake patients [61]. Only one study investigated the reliability of evoked force using ES and reported an excellent intra-investigator variability in ICU patients (*i.e.* CV = 6%) [56].

### Evoked force in ICU patients: cross-sectional and longitudinal approaches

#### Cross-sectional approach

AP Tw force production in response to MS was 40% lower in ICU patients (at a length of stay: ∼18.5 days) as compared with healthy subjects (*i.e.* 4.2 N [2.2–6.7 N] vs. 7 N [4.4–9.8 N], respectively) [13]. Electrically-evoked force recorded (within 24 h of inclusion) on the AP muscle in response to a 30 Hz stimulation train was 69% lower in ICU patients (mean MV duration: ~ 14 days) than in healthy subjects after a 2-week immobilization period to mimic disuse associated with ICU (*i.e.* 20 ± 16 *vs* 65 ± 19 N, respectively) [55]. This suggests that factors other than immobilization are involved in muscle weakness. Ankle dorsiflexors evoked force in response to ES was reduced by ~ 20% for Tw and by ~ 40% for trains of stimuli after one week of ventilation and ICU stay as compared to healthy volunteers [56].

**Fig. 3:**
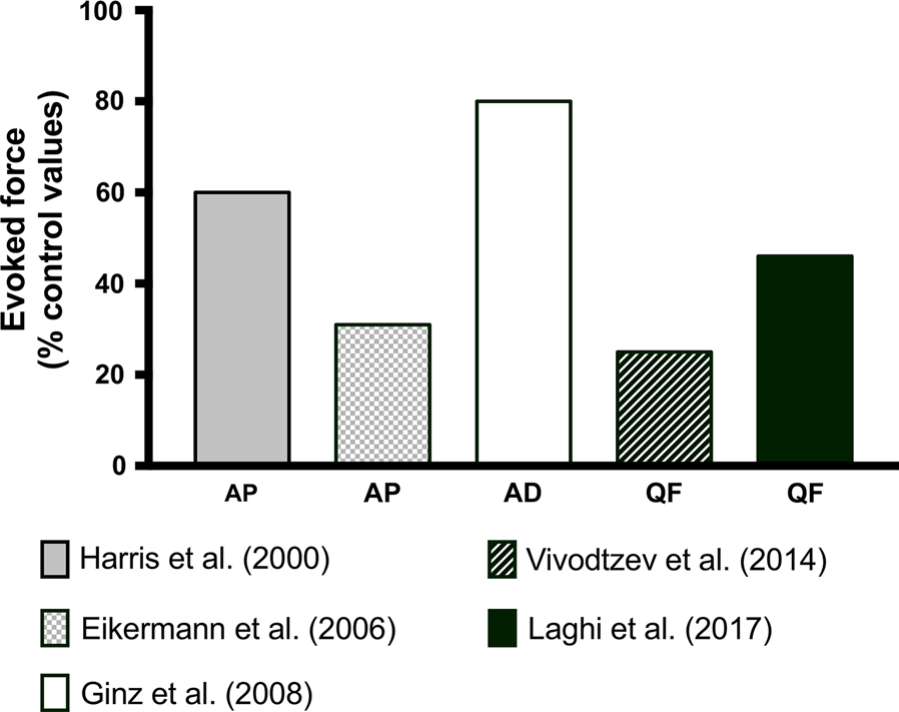
Evoked force recorded in ICU patients (expressed in percentage of control values obtained in healthy subjects) on three different muscle groups: adductor pollicis (AP), ankle dorsiflexors (AD) and quadriceps femoris (QF). Data were extracted from each respective study

Quadriceps evoked Tw force in response to MS was four times lower in ICU after a mean stay of 7 days than in healthy subjects [58, 61]. When considering quadriceps force in response to paired pulse MS stimulations, values were ~ 54% lower in ICU patients (MV duration: ~ 10 days) as compared with healthy subjects (*i.e.* 10.2 vs. 22.1 kg, respectively) [58]. Finally, the ratio between the force evoked by a tetanic stimulation at 10 Hz to the force evoked by a tetanic stimulation at 50 Hz has been found to be higher in ICU patients with sepsis than in controls [12]. The clinical relevance of this index is unclear and no information on the magnitude of muscle weakness was provided in this study. In summary, although the clinical conditions varied between these studies, ICU patients showed large reduction in evoked force in both upper and lower limb muscles (Table 4 and Fig. 3).

**Table 4:** Main outcomes from studies measuring evoked force in ICU patients

References	Muscle	Stimulation technique	Number of patients (controls)	Duration of ICU stay or MV^$^ (days [range])	Main results
Finn et al. [12]	Adductor Pollicis	ES	44 (26)	9.5 [0–38]	F10/F50^*^ ratio was higher in patients than in controls
Harris et al. [13]		ES & MS	12 (38)	18.5 [1–89]	Force was 40% lower in patients as compared with controls
Eikermann et al. [55]		ES	13 (7)	13.5 [5–23]^$^	Force was 69% lower in patients as compared with controls
Connolly et al. [15]		ES	21	13 [9–25]	Force was lower within the 24 h of admission in patients as compared with control values obtained in healthy subjects Force remained unchanged when recorded 7 days after the initial measurements
Ginz et al. [56]	Ankle Dorsiflexors	ES	19 (20)	7 [N/A]	Force was 20–40% lower in patients as compared with controls
Ginz et al. [60]		ES	8	5 [2–10]	Force decreased during the ICU stay and recovered after weaning of MV in ICU survivors
Silva et al. [14]	Quadriceps	ES^#^	30 (30)	23 [15–26]	Force decreased by ~ 25 and ~ 36% after 14 days of MV
Laghi et al. [58]		MS	12 (50)	9.9 [1–22]^$^	Force was 54% lower in patients as compared with controls
Vivodtzev et al. [61]		MS	13 (8)	7 [N/A]	Force was 75% lower in patients as compared with controls

^*^F10/F50: ratio between the forces produced by a 10 Hz stimulation train to the force produced by a 50 Hz stimulation train

^#^Electrical stimulation was applied over the quadriceps muscle belly; ES: electrical stimulation; MS: magnetic stimulation; MV: mechanical ventilation; N/A: Not available

#### Longitudinal approach

There is a paucity of studies that assessed the time-course of changes in evoked force in ICU patients. In a small cohort of 8 patients, seven of them showed a gradual decrease in electrically-evoked ankle dorsiflexor force during the ICU stay [60]. Interestingly, force significantly recovered after weaning of MV in ICU survivors but continued to decrease in the two patients who eventually died. In addition, ankle dorsiflexor force recorded within the 24 h of admission was already lower than that recorded on similarly aged healthy controls, suggesting that muscle weakness may be related to the influence of both critical illness and the presence of associated comorbidity [15]. In this study, muscle weakness was not further exacerbated when force was recorded 7 days after the initial testing. On the contrary, quadriceps electrically-evoked force decreased by ~ 25% and by ~ 36% at day 7 and day 14 of ICU stay [14], respectively.

### Limitations

Although both ES and MS techniques seem to be reliable to record evoked-force in ICU patients, these results have been obtained on small cohorts of patients (*i.e.* n < 15 patients). There is so far no-commercially available ergometer allowing for evoked-force measurements at the beside and these investigations require specialized expertise. Discomfort associated to ES may prevent longitudinal force measurements in ICU patients after awakening [12, 56]. The level of discomfort is lower when using MS but the conditions of stimulation supramaximality with MS may not be met in overweight/obese patients [62] or in patients with edema which represent a large proportion of ICU patients. It is unclear whether and to what extent force measurements on a single muscle group may be representative for generalized muscle weakness.

## Conclusions and perspectives

Over the last decade, force evaluation at patient bedside evolved from subjective to objective/quantitative measurements. Non-invasive electrical and/or magnetic evoked force measurements could be a relevant strategy to characterize muscle weakness in the early phase of ICU (*i.e.* in sedated patients). However, there is still a paucity of ergometers adapted to routine clinical practice and the reliability of evoked force measurements remains to be carefully investigated. Only a few longitudinal studies have characterized changes in evoked force from admission to awakening and/or to hospital discharge. Moreover, unlike with voluntary force measurements, the link between evoked force measurements and the diagnosis of ICUAW remains to be established. Overall, prospective multicenter ICU cohort studies are needed to determine whether and to what extent (e.g. cutoff values) evoked force measurements can be used as a valid surrogate of MRC for ICUAW diagnosis. This would allow to identify ICU patients most at risk early and will subsequently enable tailored interventional strategies, which can be delivered in the critical period to try to minimize the related alterations of neuromuscular function. Bedside ergometers could also be used to provide a comprehensive characterization of skeletal neuromuscular function in fully cooperative patients in order to get information on maximal voluntary force (as usually assessed by HHD), voluntary activation using superimposed stimuli on a maximal voluntary contraction (to evaluate whether and to what extent neural drive is impaired in ICU patients) and Tw properties (an index of muscle function) (Fig. 1) [52]. Evoked force measurements should also be combined with surface electromyography and ultrasound analyses, that can be easily performed at the bedside in sedated patients, in order to get a clear picture of the deleterious consequences of an ICU stay on the neuromuscular system and to improve our knowledge of the pathophysiology of ICUAW. The application of ES over the muscle belly (also refers to as neuromuscular ES) has been considered as a potential strategy for limiting/preventing muscle weakness/atrophy in ICU patients. However, its effectiveness is still equivocal in ICU patients [14, 63], one reason likely being methodological limitations. Indeed, the force produced in response to the stimulation, known as the main determinant of neuromuscular ES effectiveness [64], has never been accurately quantified in ICU patients. Therefore, the use of bedside ergometers could also allow to objectively quantify the individual contractile response to neuromuscular ES and identify potential responders to neuromuscular ES.

## Supplementary Information

**Additional file 1**: Search strategy used for the review

## Data Availability

Not applicable.
