# Supplementary material for: Bedside voluntary and evoked forces evaluation in intensive care unit patients: a narrative review
Source: Crit Care. 2021 Apr 22;25:157. doi: 10.1186/s13054-021-03567-9 (PMC8063302; doi:10.1186/s13054-021-03567-9)
Supplement: Supplementary file 1 — Additional file 1: Search strategy used for the review [file 13054_2021_3567_MOESM1_ESM.docx]

**Supplemental material n°1**

Articles were searched through the PubMed database (up to March 2020) using the keywords (“ICU” OR “sedated” OR “critical ill”) AND (“force” or “strength” OR “evoked force” OR “muscle stimulation” OR “electrical stimulation” OR “magnetic stimulation” OR “voluntary force” OR “handgrip” OR “handheld” OR “ergometer” OR “dynamometer”). Articles were then checked for relevant content by two authors (DK & EL) of the present review and included in the current article when investigating voluntary or non-voluntary force measurement of limb muscles in ICU patients. Further articles were considered after scanning the reference lists of the included articles. Only articles published in English were included for a total of 29 articles.
